# Perceptual learning of task-irrelevant features depends on the sensory context

**DOI:** 10.1038/s41598-019-38586-8

**Published:** 2019-02-07

**Authors:** Patrick Bruns, Takeo Watanabe

**Affiliations:** 1 Department of Cognitive, Linguistic & Psychological Sciences, Brown University, 190 Thayer Street, Providence, RI 02912 USA; 2 Biological Psychology and Neuropsychology, University of Hamburg, Von-Melle-Park 11, 20146 Hamburg, Germany

## Abstract

The brain has evolved to extract behaviourally meaningful information from the environment. For example, it has been shown that visual perceptual learning (VPL) can occur for task-irrelevant stimulus features when those features are consistently paired with internal or external reinforcement signals. It is, however, unclear whether or not task-irrelevant VPL is influenced by stimulus features that are unrelated to reinforcement in a given sensory context. To address this question, we exposed participants to task-irrelevant and subliminal coherent motion stimuli in the background while they performed a central character identification task. A specific motion direction was consistently paired with the task-targets, while two other directions occurred only with distractors and, thus, were unrelated to reinforcement. We found that the magnitude of VPL of the target-paired direction was significantly greater when the distractor-paired directions were close to the target-paired direction, compared to when they were farther. Thus, even very weak signals that are both subliminal and unrelated to reinforcement are processed and exert an influence on VPL. This finding suggests that the outcome of VPL depends on the sensory context in which learning takes place and calls for a refinement of VPL theories to incorporate exposure-based influences on learning.

## Introduction

Visual perceptual learning (VPL) is defined as a long-term improvement in the ability to perform a perceptual task as a result of perceptual experience and is regarded as a manifestation of plasticity in a perceptual system and the brain^1,2^. Such performance improvements typically occur after explicit training to distinguish or detect a particular feature^3–5^ but have also been observed after mere exposure to a feature that is irrelevant to a given task^6–11^. Theories of VPL have suggested that task-irrelevant learning is mediated by concurrent internal or external rewards. According to them, diffusive neuromodulatory signals from the reward system interact with bottom-up processing of the task-irrelevant feature, thereby helping to exceed an assumed learning threshold^12–15^. However, here we show results that cannot be explained by the theory.

The reinforcement theory of task-irrelevant VPL was based on findings showing that stimuli that are consistently paired with task-targets or rewards are learned, even in the absence of attention to these stimuli. For example, performance in a coherent motion discrimination task was found to be improved after participants were repeatedly exposed to subthreshold coherent motion stimuli in the background while performing a foveal rapid serial visual presentation (RSVP) task at the centre^10^. Crucially, task-irrelevant learning occurred only for motion directions that were temporally paired with the targets in the RSVP task, but not for motion directions that were paired with the distractors^16^. It was assumed that target recognition in the RSVP task caused an internal reward signal that drove learning of the target-paired coherent motion direction, while distractor-paired motion directions were unrelated to reward signals and consequently were not learned^12,14^. In line with this assumption, it has been shown that VPL occurs in the absence of a task when the feature is paired with an external reward^17^. These results led to the reinforcement theory in which VPL of a task-irrelevant feature occurs when the feature signal is reinforced and enhanced by reward.

The emphasis of current theories of task-irrelevant VPL on a temporal relationship between reinforcement signals and stimulus feature exposure shares similarities with the concept of classical conditioning^14,18^. In classical conditioning, it is well-known that the specificity of learning strongly depends on the presence of stimuli that are consistently not paired with a reinforcer, an effect known as discriminative conditioning^19^. For example, in animal studies using auditory fear conditioning, learning was more specific for the conditioned tone frequency when additional neutral tones of a nearby frequency were presented during the learning phase, as compared to presentation of a distant tone frequency^20^. The finding that discriminative conditioning leads to higher learning specificity suggests that learning, at least in classical conditioning, is influenced by stimulus features that are presented without temporal overlap to the reinforcement signal.

To test the influence of unrewarded stimulus features on task-irrelevant VPL, we exposed two groups of participants to task-irrelevant coherent motion stimuli while they performed a visual RSVP task in which they had to identify digits that were presented in a sequence of letters. As in previous studies^16,21,22^, a particular motion direction was consistently paired with the targets in the RSVP task, while other motion directions were only presented with the RSVP task distractors. Crucially, the distance between the target-paired and distractor-paired motion directions was systematically varied between the two groups. In the “small” group, the distractor-paired directions were close to the target-paired direction (±10°), whereas in the “large” group, they were far apart from the target-paired direction (±60°). We found that the magnitude of VPL of the target-paired direction was significantly greater when the distractor-paired directions were close to the target-paired direction, compared to when they were farther. These results suggest that even subthreshold distractor-paired directions, which should provide very weak signals, significantly influenced VPL of the target-paired direction. The reinforcement theory in its current form cannot explain these results and needs to be modified.

## Results

Performance in the RSVP digit identification task, averaged across the five training sessions, was close to ceiling in both the “small” group (*M* = 96.8% correct, *SEM* = 1.6%) and the “large” group (*M* = 97.6% correct, *SEM* = 0.6%). Accordingly, the statistical analysis showed neither significant main effects of Group, χ^2^(1) = 0.28, *p* = 0.60, or Day, χ^2^(4) = 0.17, *p* > 0.99, nor a significant interaction between the two factors, χ^2^(4) = 0.22, *p* = 0.99.

**Figure 1:**
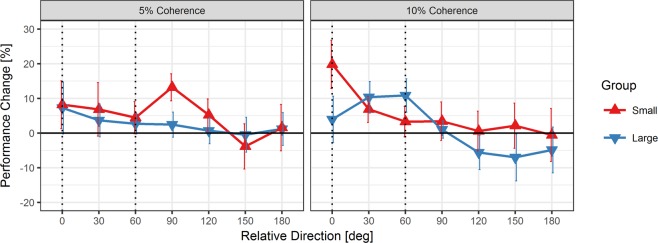
Mean performance change in motion discrimination (with *SEM*s) at 5% and 10% motion coherence in the “small” group and the “large” group. Performance change was calculated by subtracting pretest from posttest performance for each tested motion direction and then collapsing data across equivalent motion directions clockwise and counterclockwise relative to the target-paired motion direction (0°). Dotted vertical lines indicate motion directions that were paired with targets (0°, both groups) or distractors (60°, “large” group) in the RSVP task during the training sessions.

Performance in the coherent motion discrimination task was quantified as the mean normalized absolute error of participants’ motion direction judgments. Changes in performance between pre- and posttest (i.e., task-irrelevant VPL effects) are shown in Fig. 1, separately for the two groups and the two motion coherence levels that were tested (see also Supplementary Fig. S1 for a comparison of pre- and posttest performance). We focused our analysis on VPL effects at 0° (the target-paired motion direction, which was also close to the distractor-paired motion direction of ±10° in the “small” group) and ±60° (the distractor-paired motion direction in the “large” group). In the 5% coherence condition, performance in both groups was largely unchanged at posttest, except that in the “small” group there appeared to be a performance increase at ±90° (see Fig. 1). This effect, which might indicate VPL of the motion direction that was orthogonal to the target-paired direction in this group, was driven to a larger extent by two outlier values (see Supplementary Results and Discussion and Fig. S2). In the 10% coherence condition, performance improvements at posttest were primarily observed for the target-paired motion direction (0°) in the “small” group and for the distractor-paired motion directions (±60°) in the “large” group (see Fig. 1). Accordingly, a marginally significant three-way interaction between Group (“small” vs. “large”), Coherence (5% vs. 10%) and Direction (0° vs. ±60°) was obtained, *F*(1, 26) = 4.19, *p* = 0.051, ${{\rm{\eta }}}_{{\rm{P}}}^{2}$ = 0.14. Sub-ANOVAs showed that the interaction of Group and Direction was significant in the 10% coherence condition, *F*(1, 26) = 7.89, *p* = 0.009, ${{\rm{\eta }}}_{{\rm{P}}}^{2}$ = 0.23, whereas the main effects of Direction, *F*(1, 26) = 1.33, *p* = 0.260, ${{\rm{\eta }}}_{{\rm{P}}}^{2}$ = 0.05, and Group, *F* < 1, were not. By contrast, neither the interaction nor the main effects were significant in the 5% coherence condition, all *F*s < 1.

Post-hoc *t* tests for the 10% coherence condition showed that VPL effects for the target-paired motion direction (0°) were significantly larger than zero only in the “small” group, *t*(13) = 2.85, *p* = 0.027 (FDR-corrected), *d* = 0.76, but not in the “large” group, *t* < 1. By contrast, the “large” group showed a significant performance increase at ±60°, *t*(13) = 2.27, *p* = 0.041 (FDR-corrected), *d* = 0.61, whereas the “small” group did not, *t* < 1 (individual pre- vs. posttest performances in these two conditions are shown in Supplementary Fig. S3).

## Discussion

Our results challenge the reinforcement theory which assumes that task-irrelevant VPL requires a temporal relationship between exposed stimulus features and reinforcement signals^12,14^. We show that task-irrelevant VPL was influenced by subthreshold coherent motion directions that were temporally unrelated to the targets (i.e., reinforcement signals) in a visual RSVP task. VPL of the target-paired direction was significantly enhanced in the context of close compared to distant distractor-paired directions. This finding suggests that learning rules that guide classical conditioning might also guide task-irrelevant VPL. In classical conditioning, stimulus features that are unrelated to reinforcement signals influence the specificity of learning^19,20^. Similarly, in the present study task-irrelevant VPL might have been more specific for the target-paired direction when the distractor-paired directions were close but generalized to neighbouring directions when they were farther. Alternatively, participants might have learned both the target-paired and distractor-paired directions.

Previous studies have reported a substantial generalization of VPL to neighbouring directions when participants were trained on motion discrimination in a task-relevant context^23,24^. However, the present results do not appear to be fully compatible with a generalization of task-irrelevant VPL of the target-paired direction to neighbouring directions. In the “large” group, we found substantial VPL effects for the distractor-paired motion directions but no significant VPL effect for the target-paired direction itself. This finding might suggest that participants actually learned the distractor-paired motion directions instead.

**Figure 2:**
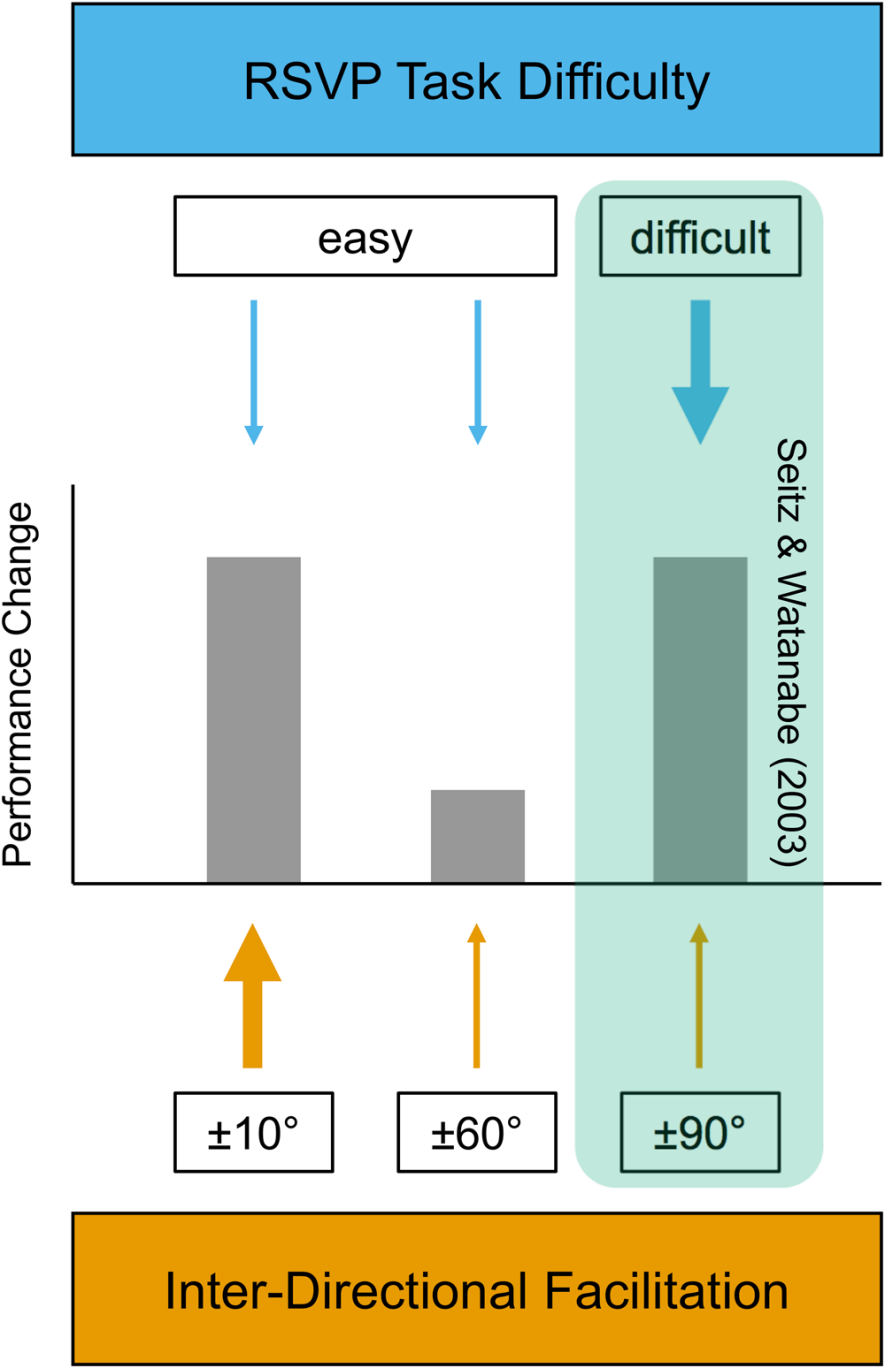
Schematic illustration of influences of two factors on performance enhancements for the target-paired motion direction in the two groups of the present study and the Seitz and Watanabe^16^ study. The strength of reinforcement signals is determined by the degree of difficulty of the central RSVP task, and the strength of inter-directional facilitation is determined by the distance between target-paired and distractor-paired motion directions.

Irrespective of whether or not task-irrelevant VPL occurred for the distractor-paired directions in our study, our results are seemingly at odds with earlier findings reported by Seitz and Watanabe^16^. In their study, in which the distractor-paired directions were 90° away, task-irrelevant VPL was specific for the target-paired direction but did not generalize to any other motion direction that was tested. However, we do not think that the present results are contradictory to the previous finding, but rather seem to provide new and additional knowledge of the mechanisms of task-irrelevant learning. Here we raise the possibility that two factors consistently account for the previous^16^ and current results (see Fig. 2). One is the strength of reinforcement signals determined by the degree of difficulty of the central RSVP task^25^, and the other is an enhancement of reinforcement-based VPL by close-by exposed directions. In the present study, in which participants had to identify two digits within a sequence of letters, RSVP task performance was close to ceiling (96.8% and 97.6% correct for the “small” and “large” group, respectively). In contrast, Seitz and Watanabe^16^ used a more demanding letter-among-letters identification task, which typically yields a performance of about 60% correct at the beginning of training and about 80% correct at the end of training^10^. It has been found that reinforcement-based task-irrelevant VPL is enhanced to a greater degree with more difficult RSVP tasks^25^. The more difficult RSVP task in the Seitz and Watanabe^16^ study may have caused stronger reinforcement signals to the target-paired direction, leading to task-irrelevant VPL of this direction. In contrast, the easier RSVP task in both groups of the present study may have provided such weak reinforcement signals that the reinforcement signals alone were not sufficiently strong to lead to task-irrelevant VPL of the target-paired direction.

Given that RSVP task performance was about the same in the “small” and “large” groups of the present study (96.8% and 97.6%, respectively), the result that a significant performance improvement for the target-paired direction was obtained only in the “small” group should be attributed to the amount of difference between the target-paired and distractor-paired directions (±10° vs. ±60°). One possibility is that reinforcement-based VPL of the target-paired direction was facilitated by close-by exposed orientations (±10°) in the “small” group. Another possibility is that reinforcement-based VPL of the target-paired-direction was inhibited by distant exposed orientations (±60°) in the “large” group. In spite of the smaller amount of reinforcement signals in the current study than in the Seitz and Watanabe^16^ study due to the easier RSVP task, task-irrelevant VPL occurred for the target-paired direction in the “small” group. Therefore, a facilitation of the target-paired direction by close-by directions in the “small” group seems more likely than an inhibition by distant directions in the “large” group. Albeit being speculative, we think that this provides a testable account of our findings, which is not necessarily incompatible with the earlier findings.

It has been argued that task-irrelevant VPL results from an interaction of diffusive neuromodulatory signals from the reward system with bottom-up processing of the task-irrelevant feature^12,14,15^. However, the distractor-paired motion directions in the present study were presented subliminally and without temporal overlap to reinforcement signals. Consequently, they should have provided very weak signals that are usually considered insufficient to drive learning^13^. Thus, neither VPL of the distractor-paired motion directions nor an influence of them on VPL of the target-paired motion direction could be explained by neuromodulatory signals that are released in response to the task targets. Theories of task-irrelevant VPL should be modified to include such purely exposure-based effects that are unrelated to task performance and reinforcement signals.

Exposure-based learning has been demonstrated in a variety of paradigms. For example, passive sensory stimulation that does not involve performance of a task or reinforcement signals has been shown to improve visual luminance change detection^6^, auditory localization accuracy^26^, and tactile acuity^27^. However, in these studies the sensory stimulation was salient and clearly perceptible, whereas learning of subliminal features has almost exclusively been reported when the stimuli were linked to reinforcement signals^16,17,28^.

What could explain changes in VPL due to coherent motion stimuli that are both subliminal *and* unrelated to reinforcement? Previous studies have shown that unattended visual features (e.g., orientation) are processed in visual cortex even when masking rendered them invisible^29,30^. Thus, it can be assumed that the distractor-paired motion directions in the present study received feature-selective cortical processing even though they were presented subliminally. Importantly, participants can automatically develop expectations for the most frequently presented directions of motion, even when they are not aware of the probability distribution^31^. Such expectations have been shown to alter perception of new motion directions by inducing biases in neural representations of motion direction in visual cortex^32^. If participants in our experiment implicitly learned the distribution of task-irrelevant motion directions used during the training sessions (either spanning ±10° or ±60°) in a similar manner, their learned prior (or expectation) of the feature statistics might have influenced their motion direction perception in the posttest. Such a mechanism would suggest that exposure-based and reinforcement-mediated VPL are separable processes that jointly determine learning outcomes. However, further studies are needed to clarify if and under which circumstances exposure-based VPL can occur in situations that do not involve reinforcement.

VPL is generally thought to occur for stimulus features that are behaviourally relevant to an organism, either because they are pertinent to a given task or because they are linked to reinforcement signals^12,14,15^. Contrary to this intuitive assumption, the results of the present study show that even very weak, subliminal signals that are unrelated to reinforcement are processed and exert an influence on VPL. This finding suggests that the outcome of VPL depends on the sensory context in which learning takes place. Theories of VPL should be refined to incorporate such exposure-based influences on learning outcomes.

## Methods

### Participants

Twenty-eight healthy adult volunteers from the Brown University community (9 males and 19 females), aged between 18 and 38 years (*M* = 22.6 years), participated in the study, which comprised seven sessions of approximately one hour each. Sessions were conducted on separate days and were completed within 9–21 days (*M* = 13 days). Participants were randomly assigned to one of two groups of 14 participants each (see below). All participants had normal or corrected-to-normal vision and were naïve regarding the purpose of the experiment. They were compensated $10 per hour for their participation. Written informed consent was obtained from all participants prior to taking part. The study was performed in accordance with the ethical guidelines laid down in the Declaration of Helsinki and the procedure was approved by the Institutional Review Board at Brown University.

### Apparatus and Stimuli

Stimulus presentation was controlled with Psychophysics Toolbox^33^ for MATLAB (The MathWorks, Natick, MA) on an Apple Mac Pro computer. All stimuli were presented on a 22” LCD monitor (ViewSonic, Walnut, CA) with a resolution of 1280 × 800 pixels and a refresh rate of 60 Hz. The viewing distance was 35 cm. A chin rest was used to maintain participants’ head position. Participants used a standard computer keyboard (training sessions) and mouse (pre- and posttest) to make responses.

Coherent motion stimuli were presented during all phases of the experiment within an invisible circular aperture (13° diameter) that was centred on the screen. Each frame (duration 16.7 ms) consisted of approximately 70 white dots with a size of 2 × 2 pixels (0.1° visual angle) each. The luminance of the dots was 80 cd/m^2^. Depending on condition, either 5% or 10% of the dots in each frame were randomly selected and replotted on the next frame in such a way that they moved in a predetermined direction at a speed of 12°/s, while the remaining dots were replotted at random locations^5,21,34^.

The background was black except for a small grey disk (1° diameter) at the centre of the display. During pre- and posttest sessions, participants were asked to fixate a bull’s eye point that was presented at the centre of the grey disk. During training sessions, capital letters and digits (approx. 0.5° visual angle) were presented in light grey Monaco font at the centre of the disk.

### Pre- and Posttest

Before (pretest) and after (posttest) the completion of five training sessions (see below), participants’ performance on a coherent motion discrimination task was measured. Pre- and posttest sessions comprised 480 trials each, which were subdivided in 10 runs. Each trial began with the presentation of a white fixation bull’s eye at the centre of the screen for 750 ms. Then, a motion stimulus was presented in the circular aperture around the central fixation disk for 500 ms. One of 12 coherent motion directions (10°, 40°, 70°, 100°, 130°, 160°, 190°, 220°, 250°, 280°, 310°, or 340°) was randomly selected and presented at one of two coherence levels (5% or 10%). Each combination of motion direction and coherence level was presented twice per run, resulting in 20 trials per condition overall.

After the offset of the motion stimulus, the colour of the fixation bull’s eye turned from white to light grey. Participants reported the perceived coherent motion direction during this light grey fixation period. As soon as they moved the mouse, a white response bar appeared that extended from the central fixation disk to the edge of the circular aperture within which the motion stimuli were presented. The orientation of the bar was controlled via mouse movements. Participants were instructed to align the orientation of the bar with the perceived coherent motion direction and confirmed their selection by clicking the mouse button (for similar procedures, see^32,35,36^). No feedback regarding response accuracy was given to participants. The response bar disappeared after the mouse click, and 750 ms later the next trial began.

### Training Sessions

The procedure of the training phase was similar to previous studies of task-irrelevant VPL^16,21,22^. Between the pre- and posttest sessions, all participants completed five training sessions in which they performed an RSVP task. Each training session comprised 400 trials, which were subdivided in 10 runs. In each trial of the RSVP task, following a 750 ms fixation period a sequence of six items (2 digits and 4 letters) was presented within the central 1° grey disk. After the offset of each sequence, participants were asked to report the two digits (which were randomly selected out of “1”, “2”, “3”, and “4”) in the order that they appeared within the sequence by pressing the corresponding number keys on a keyboard. The four letters (which were randomly selected out of “A”, “B”, “C”, “D”, “E”, “F”, “G”, “H”, “J”, “K”, “L”, “M”, “N”, “P”, “Q”, “R”, “S”, “U”, “V”, “W”, “X”, and “Y”) were distractors that could be ignored by the participants. No feedback regarding response accuracy was provided. The next trial started 750 ms after the response.

During the presentation of each digit and letter, a task-irrelevant coherent motion display was exposed in the background within the 13° circular aperture surrounding the central grey disk for 500 ms (i.e., 30 frames). The RSVP item (digit or letter) was presented together with the background motion for 366 ms (i.e., 22 frames), starting on the 5^th^ frame and disappearing on the 27^th^ frame of the motion display. Each sequence of two digits and four letters, thus, lasted 3000 ms. For each participant, one of three coherent motion directions (70°, 190° or 310°, counterbalanced across participants) was designated as target direction that was always paired with the presentation of the digits (i.e., targets) within the RSVP sequence. Two other motion directions were designated as distractor directions that were paired with the letters (i.e., distractors) in the RSVP task. Participants were randomly assigned to one of two groups. In the “small” group, the two distractor directions were close to the target direction (±10°), whereas in the “large” group, they were far apart from the target direction (±60°). Each of these two directions was presented together with two out of the four letters in each RSVP sequence. Thus, all three directions were exposed equally often during the training sessions (with the number of training sessions equated between the two groups), but only one of the three directions was paired with the targets in the RSVP task. All motion stimuli were exposed with a coherence level of 5%, which is usually close to perceptual threshold^21^ and has proven as optimal for eliciting task-irrelevant VPL effects^10,16,22^.

### Data Analysis

Responses in the RSVP digit identification task during the five training sessions were coded as either correct or incorrect. The resulting binary values were fitted using a generalized linear mixed model (GLMM), which allowed for performing a logistic regression on the data while accounting for both the between-participant factor Group (“small”, “large”) and the within-participant factor Day (1, 2, 3, 4, 5). Type III χ^2^ likelihood ratio tests were used to test for significant deviations of the estimated parameters from zero.

To quantify the change in motion discrimination performance from pre- to posttest, the absolute error (in degrees) of the selected motion direction compared to the presented coherent motion direction was determined for each trial and converted to a percentage ratio relative to chance level performance^35,36^. On average, chance level performance would result in an absolute error of 90°. Thus, 100 ×$\frac{90^\circ -|error|}{90^\circ }$ yields an accuracy measure that ranges from chance level performance (0%) to perfect estimation (100%). Note that the accuracy measure as defined above takes negative values when the selected direction is more than ±90° apart from the presented direction. Accuracy measures were averaged for each tested motion direction and coherence level (5% and 10%), separately for the pre- and posttest. Performance change was then calculated by subtracting pretest from posttest performance for each tested motion direction. The resulting values were collapsed across equivalent motion directions clockwise and counterclockwise relative to the target-paired motion direction (i.e., ±30°, ±60°, ±90°, ±120° and ±150°) and analysed.

Since previous studies have indicated that task-irrelevant VPL effects are highly specific for the trained motion directions^10,16^, we hypothesized that potential differences between the two groups would mainly emerge for the target-paired motion direction and possibly for the distractor-paired motion directions. Thus, the statistical analysis focused on VPL effects at 0° (the target-paired motion direction, which was also the tested motion direction that was closest to the distractor-paired motion direction of ±10° in the “small” group) and ±60° (which was the distractor-paired motion direction in the “large” group). Performance change values at these two directions were submitted to a three-way ANOVA with factors of Group (“small” vs. “large”), Coherence (5% vs. 10%) and Direction (0° vs. ±60°).

## Supplementary information

Supplementary Information

## Data Availability

The datasets generated during and/or analysed during the current study are available from the corresponding author on reasonable request.
