# Supplementary material for: Perceptual learning of task-irrelevant features depends on the sensory context
Source: Sci Rep. 2019 Feb 7;9:1666. doi: 10.1038/s41598-019-38586-8 (PMC6367344; doi:10.1038/s41598-019-38586-8)
Supplement: Supplementary file 1 — Supplementary Information [file 41598_2019_38586_MOESM1_ESM.pdf]

Supplementary Information

Perceptual learning of task-irrelevant features depends on the sensory context

Patrick Bruns and Takeo Watanabe

Supplementary Results and Discussion

Mean accuracies of motion discrimination in the pre- and posttest for the “small” and “large” groups are shown in Fig. S1 below. To determine the nature of the unexpected performance increase at ±90° that was observed in the 5% coherence condition for the “small” group, an outlier analysis was performed. Grubb’s test identified the two most extreme values in this condition (33.0% and 49.4% performance increase) as outliers, $U = 0.30$, $p = .020$. Excluding these two participants markedly reduced the mean performance increase at ±90° in the 5% coherence condition, but did not affect the result pattern in the 10% coherence condition (see Fig. S2 below): The interaction of Group and Direction remained significant, $F(1, 24) = 6.04$, $p = .022$, $\eta_p^2 = .20$, and the performance increase for the target-paired direction (0°) in the “small” group was still significantly larger than zero, $t(11) = 2.56$, $p = .041$ (FDR-corrected), $d = 0.74$.

Grubb’s test did not detect any significant outliers at 0° and ±60° in the 10% coherence condition, neither in the “small” group nor in the “large” group (all ps ≥ .077), suggesting that the critical interaction of Group and Direction was not driven by any extreme individual values. Individual pre- and posttest performances in these conditions are shown in Fig. S3 below. Overall, individual pretest performances had a relatively large spread, ranging from chance level performance to about 80% accuracy. Note that negative values (i.e., absolute errors larger than 90°) might indicate that some participants were able to discriminate the axis of motion above chance, but consistently pointed in the opposite direction. Importantly, however, a performance increase in the “small” group at 0° was consistently seen in all but three participants and was particularly pronounced in those participants that already performed above chance level in the pretest. By contrast, performance was clearly unchanged in the “large” group, with seven participants showing an increase and seven participants showing a decrease in performance at posttest. Individual differences in performance change between the two groups appeared less pronounced at ±60°, and were driven to a larger extent by those participants that performed at or around chance level in the pretest.

Taken together, these results show that VPL of the target-paired direction was a robust finding in the “small” group despite individual variations in the strength of the unexpected performance increase at ±90° in the 5% coherence condition. However, if there was any tendency out of the performance elevation at ±90°, one could be that in the “small” group, the motion directions encountered in the training sessions were very close to each other, and ±90° was (more or less) orthogonal to all of them. Thus, exposure might have had an unexpected effect on the orthogonal motion direction specifically for subthreshold stimuli. In contrast, the “large” group did not show this effect, presumably because they were exposed to three very different motion directions with only one of them being orthogonal to ±90°. Previous studies were similar to the “large” group and, thus, could not have observed such an effect. This possibility needs to be further examined in future research. Importantly, however, such an effect would not contradict our general hypothesis that distractor directions affect task-irrelevant VPL, but would rather confirm our hypothesis in an unexpected way.

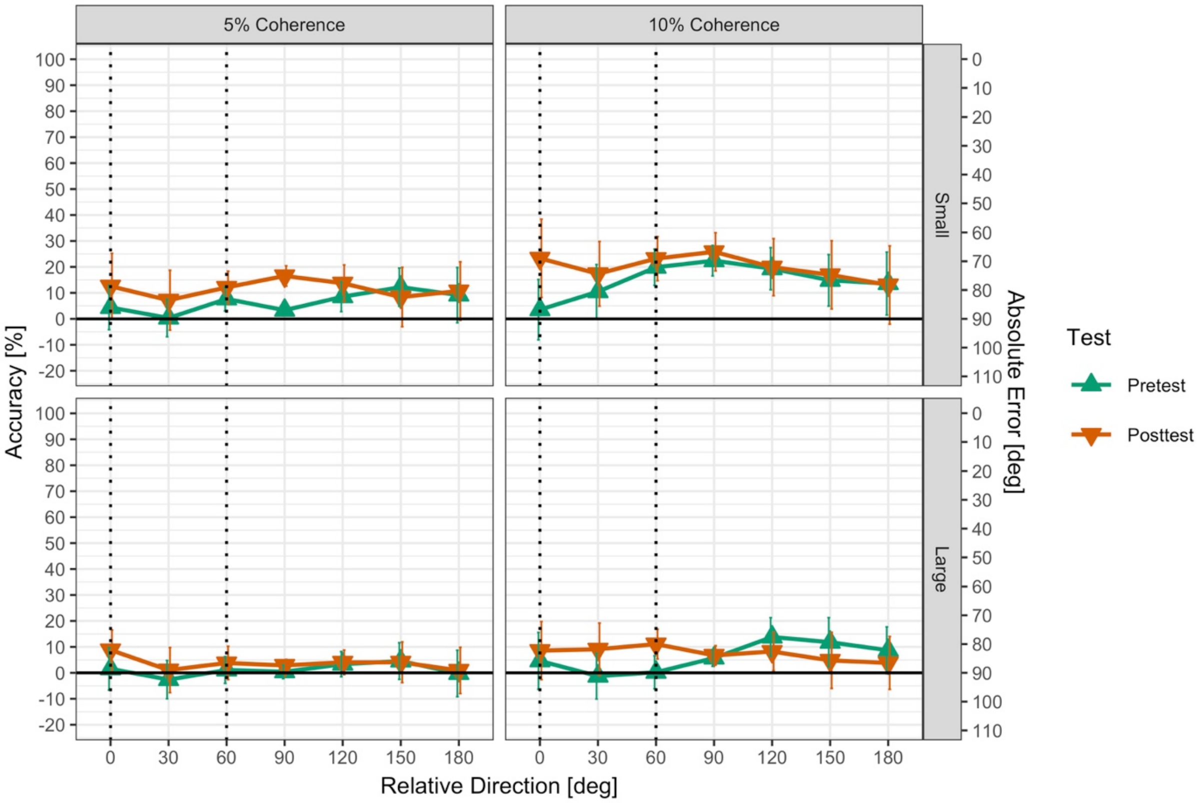

Figure S1. Mean accuracy of motion discrimination responses (with *SEM*s) for each relative motion direction before (Pretest) and after (Posttest) five training sessions. Accuracy was calculated (see Data Analysis section for details) as the percentage ratio of the absolute error of the selected motion direction relative to chance level performance (0%). For comparison, corresponding absolute error values are indicated by the second y-axis on the right. Pre- and posttest performance is shown separately for the 5% (*left column*) and 10% (*right column*) motion coherence conditions in the “small” (*top row*) and “large” (*bottom row*) groups. Dotted vertical lines indicate motion directions that were paired with targets (0°, both groups) or distractors (60°, “large” group) in the RSVP task during the training sessions.

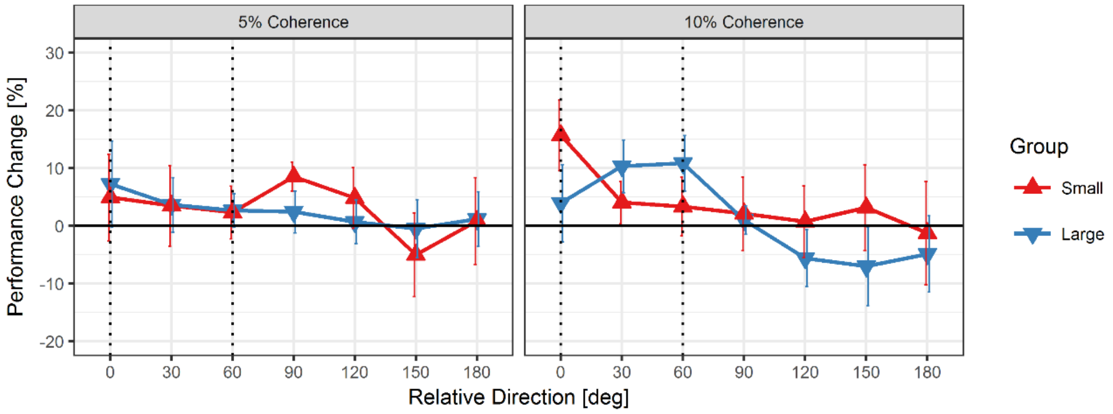

Figure S2. Mean performance change in motion discrimination (with *SEM*s) at 5% and 10% motion coherence in the “small” group and the “large” group. Same data as shown in Fig. 1, but excluding the two participants from the “small” group who showed the most extreme performance increase at ±90° in the 5% coherence condition. The amount of performance change at ±90° in the 5% coherence condition is markedly reduced in the “small” group, although other tendencies are preserved.

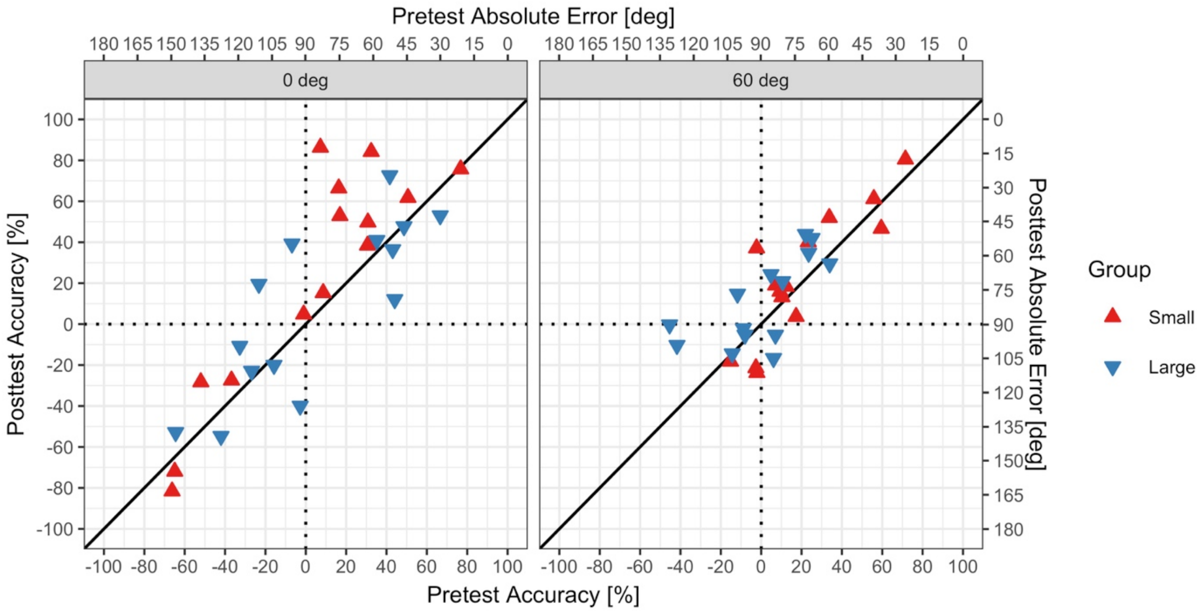

Figure S3. Individual accuracy of motion discrimination at pretest (*x*-axis) and posttest (*y*-axis) in the “small” group (n = 14) and the “large” group (n = 14). Data are shown for the 10% coherence condition, separately for the target-paired motion direction (0°) and ±60°. Second axes indicate corresponding absolute error values. The continuous line indicates the identity line and the dotted lines indicate chance level performance.
